# Annotation of gene promoters by integrative data-mining of ChIP-seq Pol-II enrichment data

**DOI:** 10.1186/1471-2105-11-S1-S65

**Published:** 2010-01-18

**Authors:** Ravi Gupta, Priyankara Wikramasinghe, Anirban Bhattacharyya, Francisco A Perez, Sharmistha Pal, Ramana V Davuluri

**Affiliations:** 1 Center for Systems and Computational Biology, Molecular and Cellular Oncogenesis Program, The Wistar Institute, Philadelphia, PA, USA; 2 Graduate Group in Genomics and Computational Biology, Department of Genetics, University of Pennsylvania, Philadelphia, PA, USA

## Abstract

**Background:**

Use of alternative gene promoters that drive widespread cell-type, tissue-type or developmental gene regulation in mammalian genomes is a common phenomenon. Chromatin immunoprecipitation methods coupled with DNA microarray (ChIP-chip) or massive parallel sequencing (ChIP-seq) are enabling genome-wide identification of active promoters in different cellular conditions using antibodies against Pol-II. However, these methods produce enrichment not only near the gene promoters but also inside the genes and other genomic regions due to the non-specificity of the antibodies used in ChIP. Further, the use of these methods is limited by their high cost and strong dependence on cellular type and context.

**Methods:**

We trained and tested different state-of-art ensemble and meta classification methods for identification of Pol-II enriched promoter and Pol-II enriched non-promoter sequences, each of length 500 bp. The classification models were trained and tested on a bench-mark dataset, using a set of 39 different feature variables that are based on chromatin modification signatures and various DNA sequence features. The best performing model was applied on seven published ChIP-seq Pol-II datasets to provide genome wide annotation of mouse gene promoters.

**Results:**

We present a novel algorithm based on supervised learning methods to discriminate promoter associated Pol-II enrichment from enrichment elsewhere in the genome in ChIP-chip/seq profiles. We accumulated a dataset of 11,773 promoter and 46,167 non-promoter sequences, each of length 500 bp, generated from RNA Pol-II ChIP-seq data of five tissues (Brain, Kidney, Liver, Lung and Spleen). We evaluated the classification models in building the best predictor and found that Bagging and Random Forest based approaches give the best accuracy. We implemented the algorithm on seven different published ChIP-seq datasets to provide a comprehensive set of promoter annotations for both protein-coding and non-coding genes in the mouse genome. The resulting annotations contain 13,413 (4,747) protein-coding (non-coding) genes with single promoters and 9,929 (1,858) protein-coding (non-coding) genes with two or more alternative promoters, and a significant number of unassigned novel promoters.

**Conclusion:**

Our new algorithm can successfully predict the promoters from the genome wide profile of Pol-II bound regions. In addition, our algorithm performs significantly better than existing promoter prediction methods and can be applied for genome-wide predictions of Pol-II promoters.

## Background

Alternative promoter usage is known to affect more than half of all human and mouse genes, and has been proposed as a primary driver of varied transcriptional regulation in different cellular conditions or developmental stages [1-4]. Numerous genes displaying complex transcriptional regulation, because of the use of alternative promoters, have been studied thoroughly [5]. Recent annotations of the human and mouse genomes suggest that many differentiation and disease-associated genes contain alternative promoters. Annotation of all human and mouse gene promoters that are differentially used during development, in different cell/tissue types or aberrantly activated in disease conditions is still incomplete and is essential for defining the transcriptome and proteome of human and mouse genomes.

The development of chromatin immunoprecipitation methods coupled with DNA microarray (ChIP-chip) technology and massively parallel sequencing (ChIP-seq) has enabled genome-wide identification of promoters, using antibody against RNA polymerase II (Pol-II) in different cells or tissues [6,7]. The combined signatures of RNA Pol-II binding and histone modification marks like H3K4me3, H3K9Ac obtained by these high throughput technologies are being used to identify human and mouse transcriptional units [8]. However, there are some challenges in predicting promoter usage based on the enrichment regions/peaks observed in these ChIP-chip/seq experiments. The ChIP-chip/seq technology requires antibodies with extremely high affinity and specificity for the target transcription factors. Unfortunately, such antibodies are not available for most human transcription factors, including Pol-II, producing non-specific enrichment in the ChIP-chip/seq profiles. The non-specific enrichment regions could be eliminated from the analysis by performing a ChIP-chip/seq experiment on the same cell or tissue lacking the specific factor. However, in most cases this is not feasible and we have to rely on other methodologies like the use of non-specific IgG ChIP-chip/seq to decrease the non-specific enrichment background. The major challenge in annotating promoters based on RNA Pol-II enriched regions/peaks is the spread of the transcribing polymerase throughout the gene and as a result all genomic regions bound by RNA Pol-II are enriched in these experiments, producing significantly large number of enriched peaks after the initial statistical analysis [9]. Though the initiator form of RNA polymerase II (phosphorylated CTD at Ser5) is enriched at a higher level in promoter region of actively transcribed genes, it is not restricted to the promoter region. Moreover, the promoters with stalled RNA Pol-II do not show an enrichment for the Ser5 phosphorylated form of RNA Pol-II [10]. Similarly, the histone marks namely H3K4me3 and H3K9Ac, which are highly enriched in promoter regions, are not exclusively present in promoter regions [8,11]. Currently it is not possible to identify promoters with high confidence based on RNA Pol-II ChIP-chip/seq enrichment data alone, thus warranting development of better classification algorithms for accurate identification of promoter related Pol-II enriched regions.

Here, we developed a computational method to discriminate promoter associated RNA Pol-II enriched regions of length 500 bp from the enrichment at other genomic regions, using the rich source of existing promoter data and associated chromatin modification signatures and various DNA sequence features. We prepared a data-set consisting of 11,773 Pol-II enriched promoters and 46,167 Pol-II enriched non-promoter regions from our recent ChIP-seq experiments, using antibody against Pol-II on five mouse tissues. We systematically trained and evaluated recent ensemble classifiers on this data set, using both 10-fold cross validation and testing on independent test set, and selected Bagging and Random Forest classifier for the final algorithm.

## Methods

### Dataset of Pol-II enriched promoters and non-promoters for training the classification models

The training set was generated from RNA Pol-II ChIP-seq data of five mouse tissues (Brain, Kidney, Liver, Lung and Spleen) generated by our lab. The RNA Pol-II ChIP-seq data was first processed and Pol-II enrichment peaks were identified at an FDR of 0.001, by assuming that peaks in the random background would follow Poisson statistics. The identified peaks were compared with TSS of non-redundant gene list generated from four different sources: RefSeq, Vega, Ensembl and UCSC. The gene lists were downloaded from UCSC genome browser [12] for mm9. Any peak that falls within -300 bp to +200 bp of known TSS (from compiled non-redundant TSS) were taken as promoter peak. The Pol-II peaks which are inside a gene but do not overlap with any of the known TSS are candidate non-promoter peaks. For our negative set, we consider only those peaks which fall within transcripts that possess a Pol-II enriched peak at the corresponding promoter (-300 bp to +200 bp). Also, any peak which falls within the promoter region of homologous gene known in other organism and within the 5' end of compiled set of non-redundant expression sequence tags (ESTs) was removed from the negative set, because some of those could be undiscovered novel promoters in the mouse genome. The homologous gene track (xenoRefGene track) was downloaded from UCSC genome browser. After identification of promoter and non-promoter peaks, the Pol-II peak was annotated as actual TSS for the transcript in that particular tissue. For each annotated peak (in both promoter and non-promoter sets), sequences were generated each of length 500 bp (-300 bp to +200 bp around the peak). After performing this procedure for all the tissues, we again compared the records in promoter and non-promoter sets with each other and removed the overlapping records from the non-promoter set. Finally, our complete dataset has 11,773 records in the promoter set and 46,167 records in the non-promoter set. We used 8,793 and 34,686 records from promoter and non-promoter sets respectively for training the classification models. In addition to 10-fold cross-validation, we used the remaining records (2,980 promoter and 11,481 non-promoter cases) to test the performance of the fitted models. The data sets are available as supplementary information at [13].

### Classification models

We tried different state-of-art ensemble and meta classifiers for identification of promoter and non-promoter classes. The WEKA data-mining toolbox [14] was used for building the classification models. The different classifiers tested on 39-dimensional feature vector are: LogitBoost [15], Bagging [16], Rotational Forest [17] and Random Forest [18]. The detailed description of the classification methods is provided in Supplementary Method.

### Feature variables used in classification model

Each sequence record (500 bp window) of the promoter and non-promoter set is represented by a 39-dimensional feature vector. The feature values were calculated based on (a) DNA sequence composition; (b) DNA physico-chemical-structural properties, and (c) experimental data. Most of the conversion tables that are based on DNA physical-chemical-structural properties were downloaded from [19]. The feature variables used in the classification models are briefly described below.

Let a given DNA sequence be: *S *= *s*_1_*s*_2_*s*_3_*s*_4_⋯*s*_*L*-1_*s*_*L*_, where *s*_*i *_∈ {*A*, *C*, *G*, *T*}, 1 ≤ *i *≤ *L *(length of sequence, here *L *= 500). Let Δ_1 _≡ {*A*, *C*, *G*, *T*}, Δ_2 _≡ {*AA*, *AC*, *AG*, *AT*, *CA*, *CC*, *CG*, *CT*, *GA*, *GC*, *GG*, *GT*, *TA*, *TG*, *TC*, *TT*}, Δ_3 _≡ {*AAA*, *AAC*, *AAG*, *AAT*, *ACA*, *ACC*, ⋯, *TTT*}, Δ_4 _≡ {*AAAA*, *AAAC*, *AAAG*, *AAAT*, *AACA*, *AACC*, ⋯, *TTTT*} represent single, di, tri, tetra nucleotide symbol set respectively.

#### (a) Properties based on DNA sequence composition

We calculate 10 different features in this category. The first 7 features are calculated from single nucleotide composition. Let *n*_*x *_represents the total number of times symbol *x *appeared in *S *and *x *∈ Δ_1 _for single nucleotide features.

1. *A_Fraction*: *n*_*A*_/L

2. *C_Fraction*: *n*_*C*_/L

3. *G_Fraction*: *n*_*G*_/L

4. *T_Fraction*: *n*_*T*_/L

5. *PurPyr_Fraction*: (*n*_*A *_+ *n*_*G *_- *n*_*C *_- *n*_*T*_)/L

6. *AmKe_Fraction*: (*n*_*A *_+ *n*_*C *_- *n*_*G *_- *n*_*T*_)/L

7. *WeSt_Fraction*: (*n*_*A *_+ *n*_*T *_- *n*_*C *_- *n*_*G*_)/L

The remaining 3 features are related to CpG island. One of the features is based on di-nucleotide composition, where *x *∈ Δ_2_. And remaining 2 features are based on tri-nucleotide composition, where *x *∈ Δ_3_. Similar CpG features were used in [19] for promoter prediction.

8. *CpG1: *(2*n_*CG *_+ 2*n_*GC*_)/(L-1)

9. *CpG2*: (*n*_*ACG *_+ *n*_*AGC *_+ *n*_*CAG *_+ *n*_*CCG *_+ *n*_*CGA *_+ *n*_*CGC *_+ 2* *n*_*CGG *_+ *n*_*CGT *_+ *n*_*CTG *_+ *n*_*GAC *_+ *n*_*GCA *_+ 2* *n*_*GCC *_+ *n*_*GCG *_+ *n*_*GCT *_+ 2* *n*_*GGC *_+ *n*_*GTC *_+ *n*_*TCG *_+ *n*_*TGC*_)/(L-2)

10. *CpG3*: (4* *n*_*CAG *_+ *n*_*CCG *_+ *n*_*CGG *_+ 4* *n*_*CTG *_+ 4* *n*_*GAC *_+ *n*_*GCC *_+ *n*_*GGC *_+ 4* *n*_*GTC*_)/(L-2)

#### (b) Properties based on physico-chemical-structural property of DNA sequences

In this category we calculate 22 features. Let *φ*_p_(*x*) represent a mapping function for a property '*P*', where *x *∈ Δ_1 _*or x *∈ Δ_2 _*or x *∈ Δ_3 _*or x *∈ Δ_4 _depending upon given property. The feature value for a given sequence '*S*' based on property '*P*' is given by , where *n*_*x *_represents total number of times symbol *x *has appeared in *S*. And Δ ≡ Δ_1 _*or *Δ ≡ Δ_2 _*or *Δ ≡ Δ_3 _*or *Δ ≡ Δ_4 _depending up on the property '*P*'.

The DNA sequence properties '*P*' from which the features are calculated is as follow:

1. A-philicity [20]

2. Base-stacking [21]

3. B-DNA twist [22]

4. DNA bending stiffness [23]

5. Di-nucleotide flexibility energy [24]

6. DNA denaturation [25,26]

7. Duplex stability disrupt energy[27]

8. Duplex stability free energy [28]

9. Helical rise [29]

10. Helical twist [29]

11. Helical tilt [29]

12. Helical roll [29]

13. Helical shift [29]

14. Helical slide [29]

15. Propeller twist [30]

16. Protein induced deformability [31]

17. Protein-DNA twist [31]

18. Z-DNA stabilizing energy [32]

19. Tri-nucleotide bendability [33]

20. Nucleosome position preference [34]

21. Tetra-nucleotide flexibility [35] and

22. EIIP [36]

#### (c) Feature variables from experimental data

In this category we calculate 7 features. The feature values are calculated from CAGE tags, RNA Pol II Chip-seq and H3K4me3 Chip-seq data sets. The CAGE tags were downloaded from FANTOM4 project [37]. RNA Pol II and H3K4me3 ChIP-seq datasets taken up for our study were downloaded from NCBI GEO website. The accession numbers for the datasets are as follow: GSE14254 [38], GSE12241 [39], GSE11074 [40], GSM281696 [41], GSM307623 [39], GSE13511 [42]. Each ChIP-seq dataset was processed at an FDR of 0.001 using our program for significant region identification. The tags which are part of significant regions were considered for feature value calculation. In total 16 different samples of H3K4me3 ChIP-seq datasets and 12 different samples of Pol-II (including 5-different tissue data generated at our lab) were collected. Total number of CAGE tag count per million (TPM) that falls in 500 bp windows was taken as CAGE tag related feature. For both, H3K4me3 ChIP-seq and RNA Pol-II ChIP-seq data we calculated the following three different features:

1. Average Tag count per million (TPM)

2. Maximum TPM

3. Maximum TPM/average TPM

The performance of the fitted models and other best performing promoter prediction programs, which are publicly available for download to run on whole chromosomes on a local computer, was tested on an independent un-seen data set. When a predicted promoter overlaps with Pol-II enriched promoter, then the respective record is counted as true positive (TP). And when such case is missed it is termed as false negative (FN). When a predicted promoter overlaps with Pol-II enriched non-promoter, then such case is counted as false positive (FP). And if such case is predicted as non-promoter then it is termed as true negative (TN). The performance of classifier is evaluated based on the promoter prediction metrics suggested by Bajic *et. al*. [43]: sensitivity (SN), positive predictive value (PPV), correlation coefficient (CC) and true-positive cost (TPC). The equations for the performance metrics are as follow:

## Results

### Classification models to predict promoters using chromatin modification signatures and DNA sequence features

**Figure 1:**
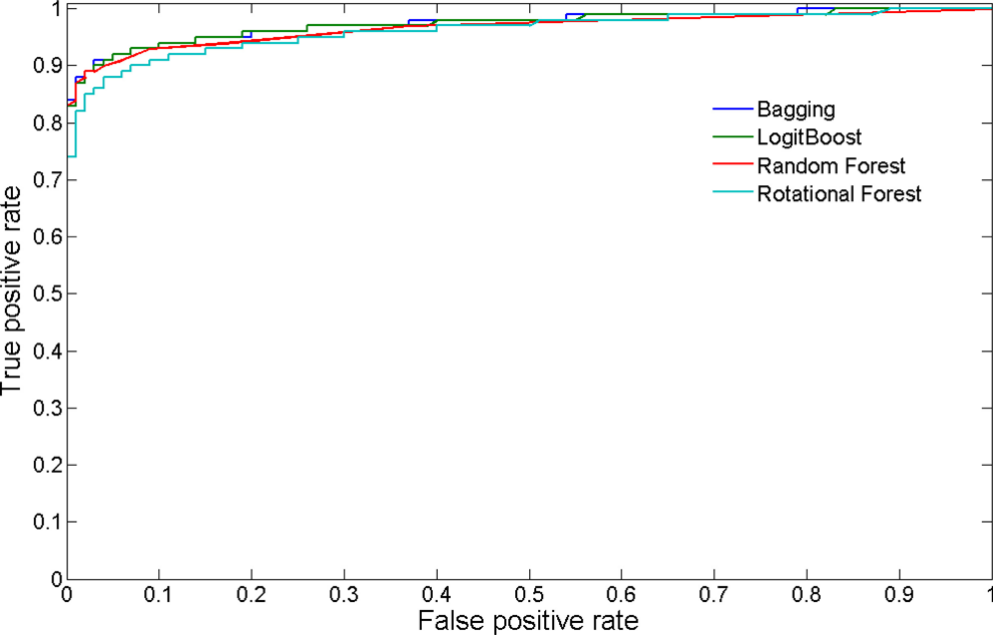
**ROC curve for four classification models**. The ROC curve obtained by 10-fold cross-validation test for the four different classification methods.

For selecting the best performing classifier, we trained four different ensemble and meta classification models on 3/4^th ^of the dataset and tested on the remaining 1/4^th ^of the dataset. The performance measures obtained by using 10-fold cross-validation and independent test set are presented in Tables 1 and 2. The performance measures, in terms of sensitivity and positive predictive accuracy, among the classifiers do not vary much over the four different models. Bagging and Random Forest models are slightly better than the other two models, showing lower error rates. Figure 1 allows for a more informative discussion on the relative predictive performance of the models. It is clear that Bagging, LogitBoost and Random Forest perform more or less similar and slightly better than Rotational Forest, with overall positive predictive value greaten than 95 and correlation coefficient greaten than 0.9. We then implemented the classification models given by Bagging and Random Forest methods in our algorithm which, will be applied to scan all the Pol-II enriched peaks in the mouse genome.

**Table 1:** Performance statistics of classification models based on 10-fold cross validation

Method	10-fold cross-validation test result, 39 features, Promoters = 8793, NonPromoters = 34686								
	
	# of true positive	# of false negative	# of true negative	# of false positive	Sensitivity (%)	Positive predictive value (PPV)	Mathew correlation coefficient	True positive cost	ROC Area
**Bagging**	7603	1190	34354	332	86.47	95.82	0.89	0.04	0.97

**LogitBoost**	7638	1155	34252	434	86.86	94.62	0.88	0.06	0.97

**Random Forest**	7626	1167	34921	395	86.73	95.08	0.89	0.05	0.97

**Rotational Forest**	7153	1640	34198	488	81.35	93.61	0.84	0.07	0.96

**Table 2:** Performance statistics of classification models and other existing programs based on independent test set

Method	Promoters = 2980, NonPromoters = 11481							
	
	# of true positive	# of false negative	# of true negative	# of false positive	Sensitivity (%)	Positive predictive value (PPV)%	Mathew correlation coefficient	True positive cost
**Bagging**	2593	387	11385	96	87.01	96.43	0.9	0.04

**LogitBoost**	2594	386	11356	125	87.05	95.4	0.89	0.05

**Random Forest**	2599	381	11349	132	87.21	95.17	0.89	0.05

**Rotational Forest**	2391	589	11332	149	80.23	94.13	0.84	0.06

**EP3 Program**	2493	487	11064	417	86.91	85.67	0.81	0.17

**Eponine Program**	2581	399	9633	1848	87.01	58.28	0.62	0.72

**ProSOM**	2563	417	8817	2664	86.01	49.03	0.53	1.04

**FirstEF**	1714	1226	11402	79	57.52	95.6	0.70	0.05

**Figure 2:**
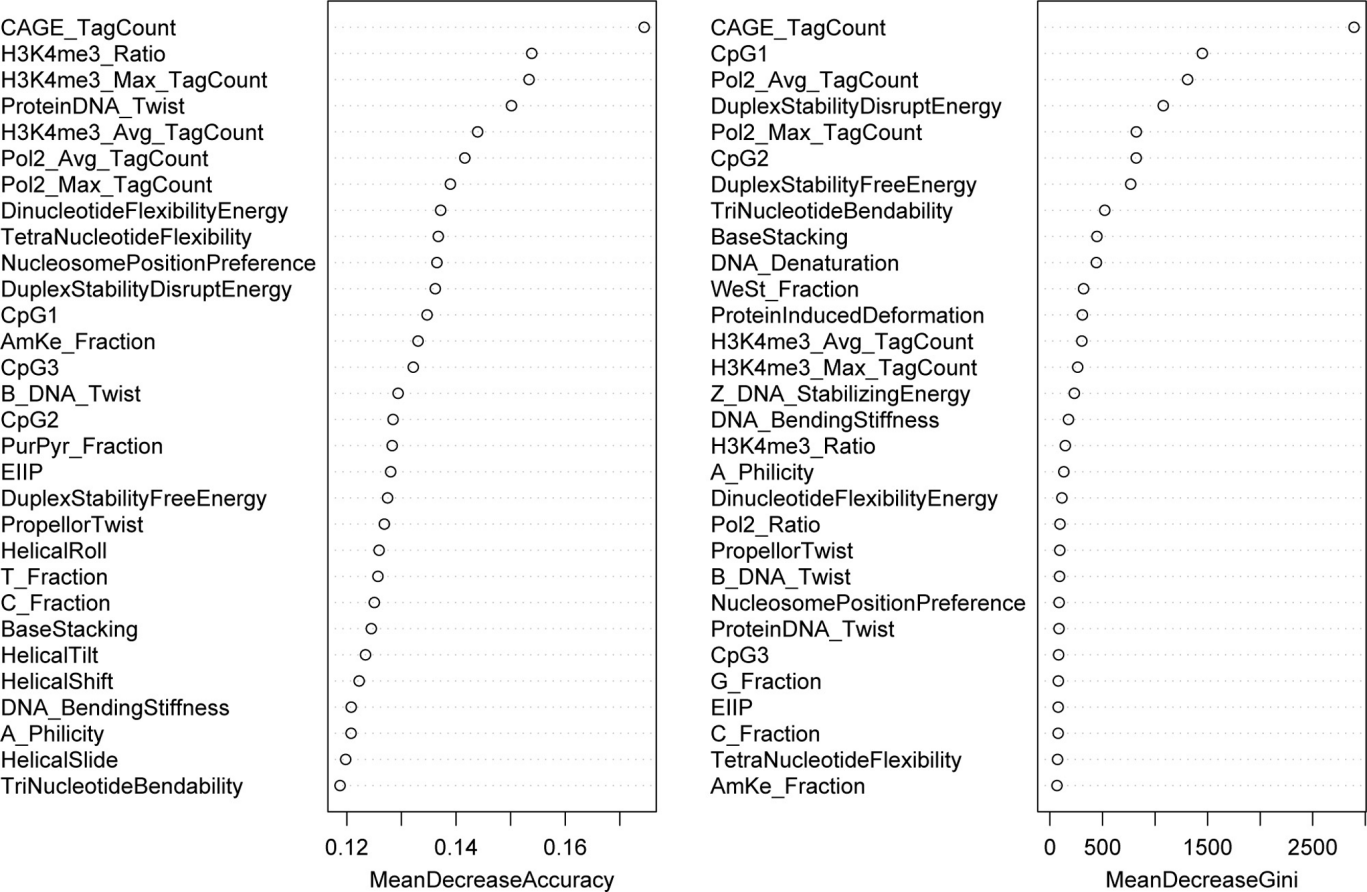
**Variable Importance table**. Top ranking feature variables selected by Random Forest and their mean decrease in accuracy and mean decrease in Gini measure in discriminating Pol-II enriched promoter regions and Pol-II enriched non-promoter regions. The mean decrease in accuracy/Gini measure was an average of 100 runs of RF.

While the classification methods used here are considered "black box" methods, with no interpretable classification model, the methods still provide useful information, such as variable importance. One of the measures of variable importance in Random Forest method is the mean decrease in accuracy, calculated using the out-of-bag sample. The difference between the prediction accuracy on the untouched out-of-bag sample and that on the out-of-bag sample permuted on one predictor variable is averaged over all trees in the forest and normalized by the standard error. This gives the mean decrease in accuracy of that particular predictor variable which has been permuted. Thus, the importance of the predictor variables can be ranked by their mean decrease in accuracy. Figure 2 shows the list of feature variables ranked according to mean decrease in accuracy of classification. It is interesting to note that feature variables based on experimental data such as CAGE tags, Pol-II enrichment, and H3K4me3 enrichment rank among the most discriminative variables from mean decrease in accuracy graph (Figure 2).

### Comparison with other promoter prediction programs

**Figure 3:**
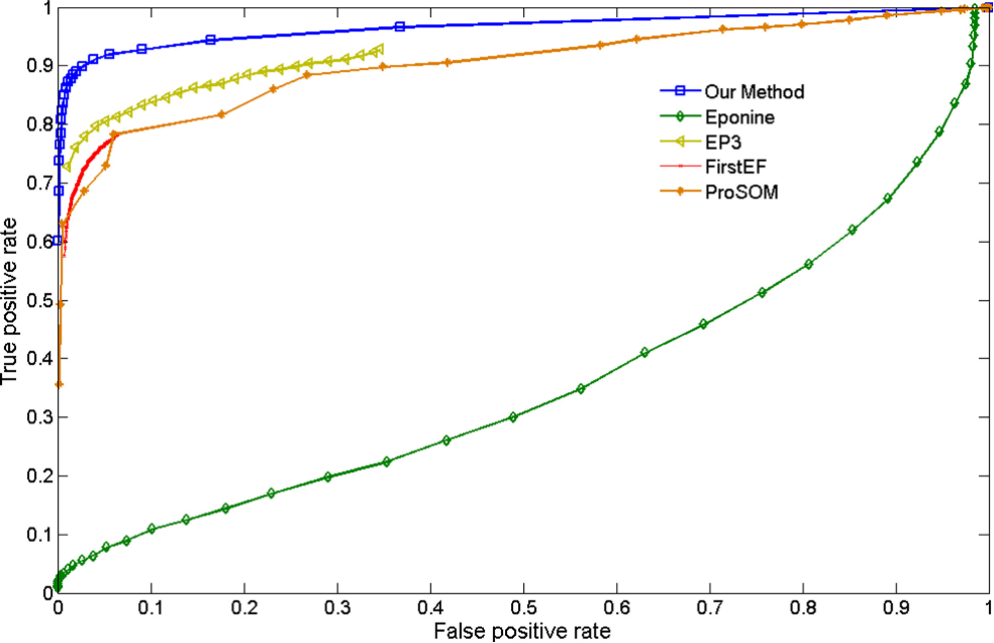
**ROC curve for comparison of our method with existing programs**. The ROC curve obtained on the test set using our method and other existing programs: EP3, Eponine, FirstEF and ProSOM.

We compared our algorithm with some of the existing best performing promoter prediction methods [44]: EP3 [19], Eponine [45], FirstEF [46], ProSOM 2.5 [47]. Table 2 and Figure 3 show that our classification model out-performs these existing programs based on independent (unseen) test set of Pol-II enriched promoters and Pol-II enriched non-promoters.

### Annotation of promoters in mouse genome using Pol-II ChIP-seq data

Although extensive promoter annotations are available from the EBI and UCSC genome servers, most annotations do not contain information about tissue or cell-type information from experimental data. To demonstrate the efficacy of our new algorithm in finding promoters and to provide the annotation of potential novel promoters, we used the Pol-II enrichment peaks obtained from seven ChIP-seq datasets available on in vitro adipocyte differentiation of mouse 3T3-L1 cells and mouse ES cells, and the results are presented in Table 3. We applied the Random forest classification model (built using the training set) on a sequence of length 500 bp around each Pol-II enriched peak (-300 to +200 bp) in both strands. If 500 bp region from both strands are predicted as peak then they are merged and counted as one promoter. For each predicted promoter region, we annotated it to a nearby gene, if the Pol-II enriched peak is located within -2 Kbp to +500 bp around the corresponding gene TSS. We generated a non-redundant TSS file for coding genes from Refseq, Vega, ensembl and UCSC genes. For non-coding genes we used information available at RefSeq, Vega, ensembl, UCSC, miRBase [48] and recently discovered large non-coding RNAs (lincRNA) [49]. Table 3 presents the total number of peaks predicted by the model as promoters and also the number of annotated coding and non-coding genes in each sample. We then combined the results from all seven samples of predicted promoters in order to identify alternative promoters for each gene. For protein coding gene set, we found that there are 13413, 5064, and 4865 genes with one promoter, two promoters, and three or more promoters respectively. In other words, based on these annotations, 42.5% of the protein coding genes in mouse genome have two or more alternative promoters. For non-coding genes, we found that 4757, 1181, and 677 genes with one promoter, two promoters, and three or more promoters respectively.

**Table 3:** Summary of prediction and annotation of Pol-II promoters from published ChIP-seq datasets

Stage	D0	D1	D2	D3	D4	D6	ES Cell
**Total tags**	5252311	5252311	5252311	5252311	5252311	5252311	2688589

**# of peaks**	108416	134674	153097	140137	159599	88606	13942

**# of peaks predicted as promoter**	24888	25179	24510	25101	22374	15838	5889

**# of predicted promoters assigned to known coding genes**	10645	10632	10349	10539	9701	8153	5034

**# of predicted promoters assigned to known non-coding genes**	1039	1095	1088	1101	1029	708	313

**# of unassigned predicted promoters (potential novel promotes)**	11684	13452	13673	13461	11644	6977	542

### Future directions

We will use this program to annotate human gene Pol-II promoters by running on all the publicly available ChIP-seq Pol-II enrichment profiles. Our method successfully predicts 500 bp promoter regions (-300 bp to + 200 bp) and to better localize the core-promoter regions within the predicted promoters, we will apply recently developed CoreBoost_HM program published by Zhang Laboratory at CSHL [50].

## Discussion

Chromatin modification and transcription factor binding profiles in the mammalian genomes is rapidly accumulating with the advent of next generation sequencing approaches. However, computational methods to effectively integrate these profiles to identify and annotate the promoter usage in specific cell/tissue types or developmental stages, are still limited. Recently, machine learning strategies have been applied to combine some of the wealth of published ChIP-seq data sets, such as chromatin modification signatures, to predict core promoter regions [50]. A logical step in analyzing the Pol-II enriched genomic regions is to scan those regions by existing promoter prediction methods to predict whether the enriched region is a promoter or non-promoter. However, we found that the performance of the existing methods is not satisfactory, and we speculate that the training set used in building the classifier was mostly responsible for their poor performance. We, therefore, build a bench-mark data set of Pol-II enriched promoters and Pol-II enriched non-promoters to train the classifiers, which shows significant improvement over the existing programs. Theoretical and empirical works using classification, regression trees, variable selection in linear and non-linear regression have shown that bagging and ensemble based methods can generate substantial prediction gain. In fact, based on the evaluation of 10-fold cross validation and testing on an independent data set, we found that both Bagging and Random Forest methods performed with highest accuracy (better than 95% prediction accuracy).

## Conclusion

In conclusion, we have developed a novel algorithm based on Bagging and Random Forest based classification methods to predict Pol-II bound promoters from ChIP-seq profiles. The present algorithm will help the discovery of novel promoters and ongoing annotation of alternative promoters of human and mouse genes from different ChIP-seq experiments.

## Supplementary material

Supplementary material is available at http://bioinfo.wistar.upenn.edu/promoterprediction/.

## Competing interests

The authors declare that they have no competing interests.

## Authors' contributions

RG designed the computational methods and performed the statistical analyses. PW performed some computational analysis, AB and FP performed database design and updates to MPromDb website. SP performed the biological experiments. RD formulated and directed the design of the study. All authors read and approved the final manuscript.

## References

[B1] Sun H Palaniswamy SK Pohar TT Jin VX Huang TH Davuluri RV MPromDb: an integrated resource for annotation and visualization of mammalian gene promoters and ChIP-chip experimental data Nucleic Acids Res 2006 34 Database D98 103 10.1093/nar/gkj096 16381984 PMC1347458

[B2] Baek D Davis C Ewing B Gordon D Green P Characterization and predictive discovery of evolutionarily conserved mammalian alternative promoters Genome Res 2007 17 2 145 155 10.1101/gr.5872707 17210929 PMC1781346

[B3] Cooper SJ Trinklein ND Anton ED Nguyen L Myers RM Comprehensive analysis of transcriptional promoter structure and function in 1% of the human genome Genome Res 2006 16 1 1 10 10.1101/gr.4222606 16344566 PMC1356123

[B4] Kawaji H Severin J Lizio M Waterhouse A Katayama S Irvine KM Hume DA Forrest AR Suzuki H Carninci P The FANTOM web resource: from mammalian transcriptional landscape to its dynamic regulation Genome Biol 2009 10 4 R40 10.1186/gb-2009-10-4-r40 19374775 PMC2688931

[B5] Davuluri RV Suzuki Y Sugano S Plass C Huang TH The functional consequences of alternative promoter use in mammalian genomes Trends Genet 2008 24 4 167 177 10.1016/j.tig.2008.01.008 18329129

[B6] Singer GA Wu J Yan P Plass C Huang TH Davuluri RV Genome-wide analysis of alternative promoters of human genes using a custom promoter tiling array BMC Genomics 2008 9 349 10.1186/1471-2164-9-349 18655706 PMC2527337

[B7] Kim TH Barrera LO Zheng M Qu C Singer MA Richmond TA Wu Y Green RD Ren B A high-resolution map of active promoters in the human genome Nature 2005 436 7052 876 880 10.1038/nature03877 15988478 PMC1895599

[B8] Barski A Cuddapah S Cui K Roh TY Schones DE Wang Z Wei G Chepelev I Zhao K High-resolution profiling of histone methylations in the human genome Cell 2007 129 4 823 837 10.1016/j.cell.2007.05.009 17512414

[B9] Rozowsky J Euskirchen G Auerbach RK Zhang ZD Gibson T Bjornson R Carriero N Snyder M Gerstein MB PeakSeq enables systematic scoring of ChIP-seq experiments relative to controls Nat Biotechnol 2009 27 1 66 75 10.1038/nbt.1518 19122651 PMC2924752

[B10] Schones DE Cui K Cuddapah S Roh TY Barski A Wang Z Wei G Zhao K Dynamic regulation of nucleosome positioning in the human genome Cell 2008 132 5 887 898 10.1016/j.cell.2008.02.022 18329373 PMC10894452

[B11] Lee BM Mahadevan LC Stability of histone modifications across mammalian genomes: implications for 'epigenetic' marking J Cell Biochem 2009 108 1 22 34 10.1002/jcb.22250 19623574

[B12] UCSC Genome Browser http://hgdownload.cse.ucsc.edu/

[B13] Center for Systems & Computational Biology, The Wistar Institute http://bioinfo.wistar.upenn.edu/promoterprediction

[B14] WEKA data-mining toolbox http://www.cs.waikato.ac.nz/ml/weka/

[B15] Friedman J Hastie T Tibshirani R Additive logistic regression: a statistical view of boosting Ann Stat 1998 28 337 407 10.1214/aos/1016218223

[B16] Breiman L Bagging predictors Mach Learn 1996 24 2 123 140

[B17] Rodriguez JJ Alonso CJ Kuncheva LI Rotation Forest: A New Classifier Ensemble Method IEEE Trans Pattern Anal Mach Intell 2006 28 10 1619 1630 10.1109/TPAMI.2006.211 16986543

[B18] Breiman L Random Forests Mach Learn 2001 45 1 5 32 10.1023/A:1010933404324

[B19] Abeel T Saeys Y Bonnet E Rouze P Peer Y Van De Generic eukaryotic core promoter prediction using structural features of DNA Genome Research 2008 18 2 310 323 10.1101/gr.6991408 18096745 PMC2203629

[B20] Ivanov VI Minchenkova LE [The A-form of DNA: in search of the biological role] Mol Biol (Mosk) 1994 28 6 1258 1271 7885327

[B21] Ornstein RL Rein R Breen DL Macelroy RD OPTIMIZED POTENTIAL FUNCTION FOR CALCULATION OF NUCLEIC-ACID INTERACTION ENERGIES .1. BASE STACKING Biopolymers 1978 17 10 2341 2360 10.1002/bip.1978.360171005 24624489

[B22] Gorin AA Zhurkin VB Olson WK B-DNA twisting correlates with base-pair morphology J Mol Biol 1995 247 1 34 48 10.1006/jmbi.1994.0120 7897660

[B23] Sivolob AV Khrapunov SN Translational positioning of nucleosomes on DNA: the role of sequence-dependent isotropic DNA bending stiffness J Mol Biol 1995 247 5 918 931 10.1006/jmbi.1994.0190 7723041

[B24] Packer MJ Dauncey MP Hunter CA Sequence-dependent DNA structure: dinucleotide conformational maps J Mol Biol 2000 295 1 71 83 10.1006/jmbi.1999.3236 10623509

[B25] Blake RD Delcourt SG Thermal stability of DNA Nucleic Acids Res 1998 26 14 3323 3332 10.1093/nar/26.14.3323 9649614 PMC147704

[B26] Blake RD Bizzaro JW Blake JD Day GR Delcourt SG Knowles J Marx KA SantaLucia J Jr Statistical mechanical simulation of polymeric DNA melting with MELTSIM Bioinformatics 1999 15 5 370 375 10.1093/bioinformatics/15.5.370 10366657

[B27] Breslauer KJ Frank R Blocker H Marky LA Predicting DNA duplex stability from the base sequence Proc Natl Acad Sci USA 1986 83 11 3746 3750 10.1073/pnas.83.11.3746 3459152 PMC323600

[B28] Sugimoto N Nakano S Yoneyama M Honda K Improved thermodynamic parameters and helix initiation factor to predict stability of DNA duplexes Nucleic Acids Res 1996 24 22 4501 4505 10.1093/nar/24.22.4501 8948641 PMC146261

[B29] Goni JR Perez A Torrents D Orozco M Determining promoter location based on DNA structure first-principles calculations Genome Biol 2007 8 12 R263 10.1186/gb-2007-8-12-r263 18072969 PMC2246265

[B30] el Hassan MA Calladine CR Propeller-twisting of base-pairs and the conformational mobility of dinucleotide steps in DNA J Mol Biol 1996 259 1 95 103 10.1006/jmbi.1996.0304 8648652

[B31] Olson WK Gorin AA Lu XJ Hock LM Zhurkin VB DNA sequence-dependent deformability deduced from protein-DNA crystal complexes Proc Natl Acad Sci USA 1998 95 19 11163 11168 10.1073/pnas.95.19.11163 9736707 PMC21613

[B32] Ho PS Zhou GW Clark LB Polarized electronic spectra of Z-DNA single crystals Biopolymers 1990 30 1-2 151 163 10.1002/bip.360300115 2224047

[B33] Brukner I Sanchez R Suck D Pongor S Sequence-dependent bending propensity of DNA as revealed by DNase I: parameters for trinucleotides EMBO J 1995 14 8 1812 1818 7737131 10.1002/j.1460-2075.1995.tb07169.x PMC398274

[B34] Satchwell SC Drew HR Travers AA Sequence periodicities in chicken nucleosome core DNA J Mol Biol 1986 191 4 659 675 10.1016/0022-2836(86)90452-3 3806678

[B35] Packer MJ Dauncey MP Hunter CA Sequence-dependent DNA structure: tetranucleotide conformational maps J Mol Biol 2000 295 1 85 103 10.1006/jmbi.1999.3237 10623510

[B36] Cosic I Macromolecular bioactivity: is it resonant interaction between macromolecules?--Theory and applications IEEE Trans Biomed Eng 1994 41 12 1101 1114 10.1109/10.335859 7851912

[B37] FANTOM4 Project http://fantom.gsc.riken.jp/4/

[B38] Wei G Wei L Zhu J Zang C Hu-Li J Yao Z Cui K Kanno Y Roh TY Watford WT Global mapping of H3K4me3 and H3K27me3 reveals specificity and plasticity in lineage fate determination of differentiating CD4+ T cells Immunity 2009 30 1 155 167 10.1016/j.immuni.2008.12.009 19144320 PMC2722509

[B39] Mikkelsen TS Ku M Jaffe DB Issac B Lieberman E Giannoukos G Alvarez P Brockman W Kim TK Koche RP Genome-wide maps of chromatin state in pluripotent and lineage-committed cells Nature 2007 448 7153 553 560 10.1038/nature06008 17603471 PMC2921165

[B40] Mikkelsen TS Hanna J Zhang X Ku M Wernig M Schorderet P Bernstein BE Jaenisch R Lander ES Meissner A Dissecting direct reprogramming through integrative genomic analysis Nature 2008 454 7200 49 55 10.1038/nature07056 18509334 PMC2754827

[B41] Meissner A Mikkelsen TS Gu H Wernig M Hanna J Sivachenko A Zhang X Bernstein BE Nusbaum C Jaffe DB Genome-scale DNA methylation maps of pluripotent and differentiated cells Nature 2008 454 7205 766 770 18600261 10.1038/nature07107 PMC2896277

[B42] Nielsen R Pedersen TA Hagenbeek D Moulos P Siersbaek R Megens E Denissov S Borgesen M Francoijs KJ Mandrup S Genome-wide profiling of PPARgamma:RXR and RNA polymerase II occupancy reveals temporal activation of distinct metabolic pathways and changes in RXR dimer composition during adipogenesis Genes Dev 2008 22 21 2953 2967 10.1101/gad.501108 18981474 PMC2577787

[B43] Bajic VB Tan SL Suzuki Y Sugano S Promoter prediction analysis on the whole human genome Nat Biotechnol 2004 22 11 1467 1473 10.1038/nbt1032 15529174

[B44] Abeel T Peer Y Van de Saeys Y Toward a gold standard for promoter prediction evaluation Bioinformatics 2009 25 12 i313 320 10.1093/bioinformatics/btp191 19478005 PMC2687945

[B45] Down TA Hubbard TJ Computational detection and location of transcription start sites in mammalian genomic DNA Genome Res 2002 12 3 458 461 10.1101/gr.216102 11875034 PMC155284

[B46] Davuluri RV Grosse I Zhang MQ Computational identification of promoters and first exons in the human genome Nat Genet 2001 29 4 412 417 10.1038/ng780 11726928

[B47] Abeel T Saeys Y Rouze P Peer Y Van de ProSOM: core promoter prediction based on unsupervised clustering of DNA physical profiles Bioinformatics 2008 24 13 i24 31 10.1093/bioinformatics/btn172 18586720 PMC2718650

[B48] Griffiths-Jones S Saini HK van Dongen S Enright AJ miRBase: tools for microRNA genomics Nucleic Acids Res 2008 36 Database D154 158 17991681 10.1093/nar/gkm952 PMC2238936

[B49] Guttman M Amit I Garber M French C Lin MF Feldser D Huarte M Zuk O Carey BW Cassady JP Chromatin signature reveals over a thousand highly conserved large non-coding RNAs in mammals Nature 2009 458 7235 223 227 10.1038/nature07672 19182780 PMC2754849

[B50] Wang X Xuan Z Zhao X Li Y Zhang MQ High-resolution human core-promoter prediction with CoreBoost_HM Genome Res 2009 19 2 266 275 10.1101/gr.081638.108 18997002 PMC2652208

